# Chromosomal *dif* sites and associated modules identified in *Acinetobacter* sp. drive the horizontal transfer of antibiotic resistance

**DOI:** 10.3389/fmicb.2025.1708097

**Published:** 2026-02-02

**Authors:** Qing Wang, Weiwei Wang, Yanhua Qiu, Guonian Dai, Bing Li, Yaxin Zhou, Yubin Bai, Jiyu Zhang

**Affiliations:** 1 College of Veterinary Medicine, Gansu Agricultural University, Lanzhou, Gansu, China; 2 Lanzhou Institute of Husbandry and Pharmaceutical Sciences, Chinese Academy of Agricultural Sciences, Lanzhou, Gansu, China; 3 Key Laboratory of New Animal Drug Project of Gansu Province, Lanzhou, Gansu, China; 4 Key Laboratory of Veterinary Pharmaceutical Development, Ministry of Agriculture and Rural Affairs, Lanzhou, Gansu, China

**Keywords:** *Acinetobacter*, antibiotic resistance genes, *dif* modules, p*dif* modules, XerCD site-specific recombination system

## Abstract

**Introduction:**

Modules containing antibiotic resistance genes (ARGs) flanked by Xer site-specific recombination sites have been identified in *Acinetobacter* plasmids and are considered mobile genetic elements (MGEs) that facilitate horizontal gene transfer via the XerCD site-specific recombination (XerCD SSR) system. Although additional *dif*-like sites have been identified on the *Acinetobacter* chromosome beyond the main locus, it remains unclear whether these sites are associated with chromosomal *dif* modules.

**Methods:**

MacConkey agar plates supplemented with meropenem were used to isolate the resistant strain. Whole-genome sequencing (WGS) was performed on the Oxford Nanopore platform, and the bacterial species was identified using Average Nucleotide Identity (ANI) and digital DNA-DNA hybridization (dDDH). Antimicrobial susceptibility testing was performed against 18 antibiotics. Identification of *dif* and p*dif* sites was performed using BLAST tools.

**Results:**

This study identified numerous Xer modules containing resistance genes, IS elements, and other functional genes within the chromosome and plasmid of strain M10 (*Acinetobacter* sp.) isolated from a farmer at a cattle farm in Guangxi, China. Genome analysis and antimicrobial susceptibility testing confirm the association between these modules carrying resistance genes and resistant phenotypes. Chromosomal *dif* sites and associated *dif* modules in the strain were highly similar (sequence identity >99%) to plasmid-carried p*dif* sites and associated p*dif* modules in the public database. These suggest that additional chromosomal *dif*-like sites facilitate *dif* module formation, and that gene flow occurs between the chromosomes and plasmids of *Acinetobacter*. Furthermore, most Xer sites clustered to form a linear multi-module array, termed chromosomal *dif* module island and plasmid-borne p*dif* module island. Similar configurations were frequently observed in public *Acinetobacter* plasmid genomes.

**Discussion:**

Additional *dif*-like sites are present in *Acinetobacter* chromosomes, which are unlikely to play a function in chromosomal dimer resolution, and the modules they form are functionally similar to p*dif* modules, both of which play an important role in horizontal gene transfer.

## Introduction

*Acinetobacter* is known for its ability to spread through food production, processing, and storage; through the hands of healthcare personnel; and through cross-contamination of medical devices, making it a significant contributor to nosocomial infections (Rathinavelu et al., 2003; De Amorim and Nascimento, 2017). The rise of antimicrobial resistance in *Acinetobacter* is a critical medical challenge. Although research on the transfer of resistance genes via canonical MGEs (such as plasmids, ICEs, and transposons) in *Acinetobacter* remains limited (Pérals et al., 2000; Wang et al., 2025), an untypical class of mobile genetic elements whose transposition likely depends on the action of the XerCD site-specific recombination (XerCD SSR) system was recently discovered in *Acinetobacter*. This system consists of the homologous recombinases XerC/XerD (tyrosine recombinase family) (Lin et al., 2020; Balalovski and Grainge, 2020), which catalyze two sequential pairs of DNA strand cuts and exchanges at a defined locus termed *dif*, located in the terminus region of the chromosome (Balalovski and Grainge, 2020; Grosso et al., 2012; Poirel and Nordmann, 2006; Midonet and Barre, 2014). Furthermore, this system is critical for stabilizing plasmids by resolving multimers (Colloms et al., 1998; Summers et al., 1993). Typically, the *dif* site is a 28-bp site consisting of two inverted repeat 11-bp Xer-binding motifs (the left and right regions) separated by a 6-bp interval called the central region (Lin et al., 2020; Balalovski and Grainge, 2020). A monomer of XerC and XerD each binds to a 11-bp semi-binding site (Balalovski and Grainge, 2020; Shao et al., 2023). The *dif* sites located in *Acinetobacter* plasmids are called p*dif* sites and appear multiple times in a plasmid (Shao et al., 2023). Modules containing ARGs flanked by *pdif* sites have been identified. Substantial copies of these modules are widely distributed across plasmid genomes within the *Acinetobacter* genus (Grosso et al., 2012; Poirel and Nordmann, 2006; Shao et al., 2023; Cameranesi et al., 2018; Merino et al., 2010; Blackwell and Hall, 2017; Tran et al., 2012), suggesting horizontal gene transfer mediated by p*dif* modules. In addition to ARGs, genes encoding other functional proteins and proteins of unknown function are carried by p*dif* modules, such as p*dif-ser,* p*dif-*IS*Ajo2*, p*dif*-*higA*-*higB*, p*dif-chrAB*, p*dif-kup*, p*dif-terC*, p*dif-add*, p*dif-sulP*, and p*dif-ohr* (Blackwell and Hall, 2017; Harmer et al., 2023; Mindlin et al., 2019). Meanwhile, the site-specific recombination reaction can be observed between the two sites in different plasmids (Cameranesi et al., 2018; Mindlin et al., 2019), further suggesting that the p*dif* module is involved in horizontal genetic transfer. However, modules containing various genes flanked by chromosomal *dif* sites are rarely observed. Usually, a single *dif* site (the main *dif* site) is involved in dimeric chromosome resolution (Mindlin et al., 2019; Carnoy and Roten, 2009). This site is considered not to participate in *dif* module formation, as its involvement would impair the dimer resolution process (Mindlin et al., 2019). Thus, in addition to the main *dif* site, additional *dif*-like sites may exist that could contribute to *dif* module formation. This study found numerous Xer sites in the chromosome and plasmid of strain M10. The presence of multiple chromosomal *dif*-like sites suggests that some may serve functions beyond canonical dimer resolution.

## Results

### Source

In September 2023, we isolated a meropenem-unsusceptible strain, M10, from the feces of a farmer at a commercial bovine farm in Guangxi, China. PCR amplification and Sanger sequencing confirmed that this strain carried the *bla*_NDM_ gene.

### Identification of bacterial species

This strain was classified as *Acinetobacter* genus using MALDI-TOF-MS and 16S rDNA. Subsequently, this strain was sequenced using whole-genome sequencing (WGS) on the Oxford Nanopore platform (long-read sequencing technology). The analysis confirmed 98.56% completeness and 0.83% contamination in this genome assembly (Acinetobacter towneri genome assembly ASM4857284v1 – NCBI – NLM). To confirm the species of this strain, the average nucleotide identity (ANI) match was performed using the NCBI annotation service on the NCBI database. This result showed that the strain best matched *Acinetobacter towneri* DSM 14962 = CIP 107472 (GCA_000368785.1), with an ANI value of 93.82%. Subsequently, these strains were submitted to the genome-to-genome distance calculator (GGDC) on the DSMZ (Deutsche Sammlung von Mikroorganismen und Zellkulture) platform to calculate the digital DNA–DNA hybridization (dDDH) value. This result demonstrated a dDDH value of 53.3%. Current standards require ANI (>95% for the same species) or digital DNA–DNA hybridization (dDDH) (>70%) values to assign a novel strain to a species robustly (Riesco and Trujillo, 2024; Meier-Kolthoff et al., 2013). These results indicate that strain M10 falls below the standard ANI (>95%) and dDDH (>70%) thresholds for assignment to an existing species. In view of this, this strain was submitted to the JSpeciesWS and DSMZ platforms using the Tetra Correlation Search (TCS) and Type (Strain) Genome Server (TYGS) for further analysis. These results were similar to those in the NCBI annotation service. Thus, genome M10 should be considered a potential novel species within the *Acinetobacter* genus.

### Phylogenetic analysis

**Figure 1:**
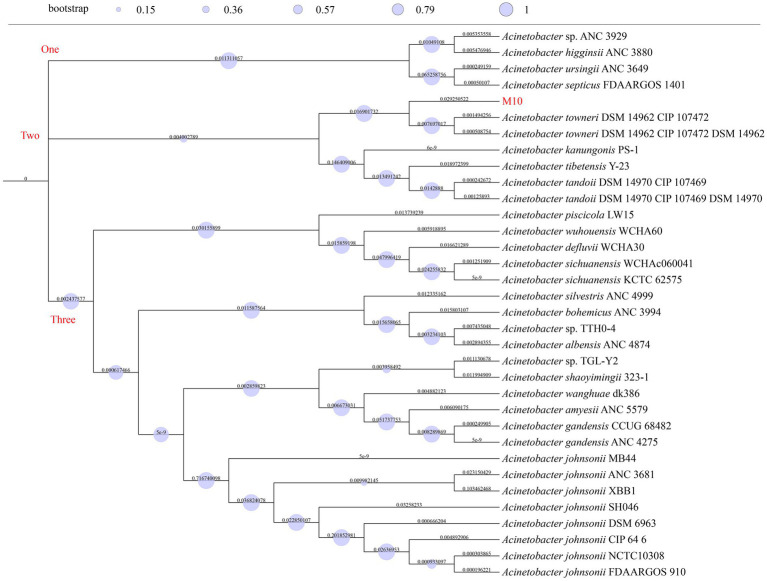
Phylogenetic tree. Bootstrap values are shown on the branch of this tree as circles. Branch lengths are displayed as numbers on each branch of this tree.

We retrieved 33 genomes closely related to strain M10 from the JSpesiesWS (TCS) and DSMZ platforms (TYGS). Subsequently, all genomes were used to build an ML phylogenetic tree (Figure 1). Phylogenetic analysis revealed that the tree diverged into three branches, highlighting three distinct lineages. Branch two included strain M10, *A. towneri* (DSM 14962 CIP 107472 and DSM 14962 CIP 107472 DSM 14962), *A. kanungonis* (PS-1), *A. tibetensis* (Y-23), and *A. tandoii* (DSM 14970 CIP 107469 and DSM 14970 CIP 107469 DSM14970). Among these, strain M10 and *A. towneri* belonged to a clade, showing their highest genetic similarity. Branch lengths in phylogenetic trees serve as important indicators for measuring genetic differences among strains. The genetic difference in strain M10 significantly exceeds that in *A. towneri* [branch length: 0.029 (M10) > 0.0077 (*A. towneri*)], indicating genetic similarity between them, though they belong to different lineages.

### Antibiotic resistance phenotype

The antimicrobial susceptibility testing showed that strain M10 was resistant to meropenem (16 mg/L), ampicillin (128 mg/L), ceftazidime (>1,024 mg/L), cefepime (128 mg/L), gentamicin (16 mg/L), tetracycline (16 mg/L), imipenem (16 mg/L), and ertapenem (64 mg/L) (Table 1). However, it was susceptible to aztreonam, chloramphenicol, colistin, kanamycin, fosfomycin, ciprofloxacin, sulfamethoxazole, azithromycin, tigecycline, and amikacin (Table 1).

**Table 1:** MIC and ARGs of the strain M10.

Antibiotics	MIC (mg/L)	Interpretation	ARGs
Meropenem (MEM)	16	Resistant	*bla*_NDM-1_, *bla*_OXA-58_
Aztreonam (ATM)	8	Susceptible	–
Ampicillin (AMP)	128	Resistant	*bla*_NDM-1_, *bla*_OXA-58_
Ceftazidime (CAZ)	>1,024	Resistant	*bla*_NDM-1_
Cefepime (FEP)	128	Resistant	*bla*_NDM-1_
Gentamicin (GEN)	16	Resistant	*aac(3)-IId*
Chloramphenicol (CHL)	16	Susceptible	–
Colistin (CL)	<2	Susceptible	–
Kanamycin (KAN)	32	Susceptible	–
Fosfomycin (FOS)	128	Susceptible	–
Ciprofloxacin (CIP)	2	Susceptible	–
Sulfamethoxazole (SXT)	256	Susceptible	–
Azithromycin (AZM)	8	Susceptible	*msr*(E)
Tetracycline (TET)	16	Resistant	*tet*(39)
Tigecycline (TGC)	<2	Susceptible	–
Imipenem (IPM)	16	Resistant	*bla*_NDM-1_, *bla*_OXA-58_
ertapenem (ETP)	64	Resistant	*bla*_NDM-1_
Amikacin (AN)	16	Susceptible	*aph(3′)-VI*
Antibiotics not included in this experiment	NA	NA	*aadA2b, mph*(E)

–: without known resistance genes in this study, NA: not applicable.

### Genetic diversity analysis

ONT long-read sequencing revealed that strain M10 carried a chromosome (chromosome-NDM-M10) and a plasmid (pM10), with sizes of 2,896,975 bp and 162,635 bp (Table 2). The chromosome-NDM-M10 harbored multiple ARGs, including *aadA2b*, *aac(3)-IId, tmrB*, *aph(3′)-VIa*, *bla*_NDM-1_, *ble*_MBL_, *bla*_OXA-58_, *msr*(E), *mph*(E), and *tet*(39) (Table 2). In addition, the chromosome carried metal resistance genes (MRGs) against arsenic, mercury, and copper, as well as genes encoding virulence factors (VFs) related to bacterial outer membrane protein, type IV pili, phospholipase D, LPS, two-component system, and serum resistance (Table 2). The pM10 carried three ARGs, including *msr*(E), *mph*(E), and *tet*(39) (Table 2). The analysis using MOB-Typer revealed that pM10 could be typed as rep_cluster_1656 and predicted to be mobilizable.

**Table 2:** Genomic characteristics of the strain M10.

M10	Inc	Size (bp)	GC content	ARGs	MRGs	VF
Chromosome-NDM-M10	–	2,896,975	41.2%	*aadA2b, aac(3)-IId, tmrB, aph(3′)-VIa, bla*_NDM-1_*, ble_MBL_, bla*_OXA-58_*, msr*(E)*, mph*(E)*, tet*(39)	Arsenic: *arsH*	Outer membrane protein: *ompA* Type IV pili: *pilE* Phospholipase D: *plcD* LPS: *lpsABCDLM* Two-component system: *bfmRS* serum resistance: *pbpG*
					Mercury: *merACR*	
					Copper: *copAB*, *czcABDO*	
pM10	rep_cluster_1656	162,635	39.5%	*msr*(E)*, mph*(E)*, tet*(39)	–	–

–: without known genes in this study. VF: virulence factors associated genes.

### Chromosomal *dif* sites

**Figure 2:**
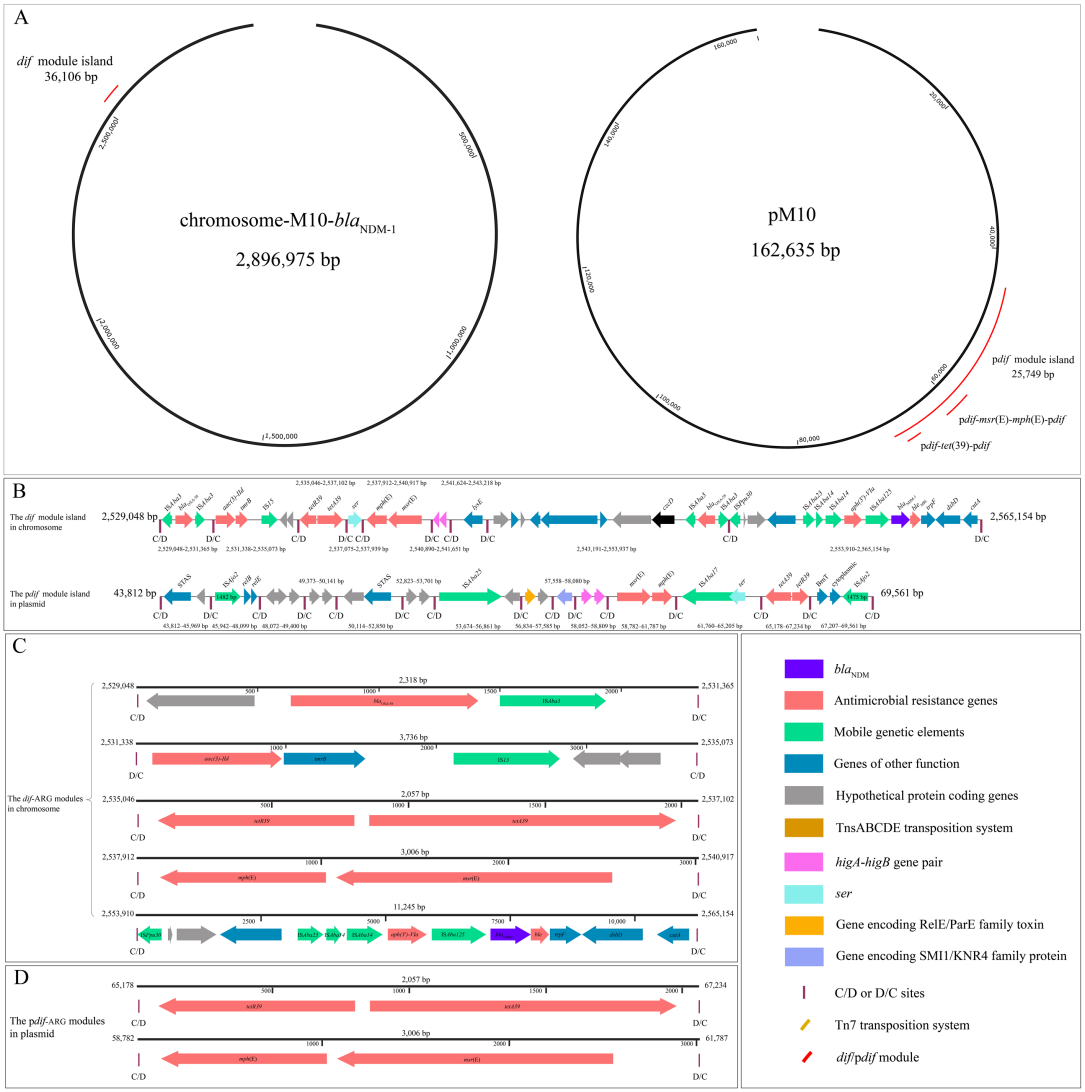
The show of *dif* and p*dif* modules and the islands they build. **(A)** The locations of Xer module islands are shown in the circular genome of the chromosome and plasmid. **(B)** Linear environment of the *dif* and p*dif* module islands. **(C)** Linear skeletons of *dif* modules. **(D)** Linear skeletons of *pdif* modules. Vertical bars indicate *dif* or p*dif* (C/D and D/C) sites; the direction of each site is shown.

A total of 22 *dif* sites were identified in the chromosome of strain M10. Among these, one *dif* site was identified as the main *dif* site (1,434,250–1,434,277 bp). The remaining *dif* sites likely facilitate the formation of *dif* modules. Notably, ten *dif* sites clustered in a region of 36,106 bp (2,529,048–2,565,154 bp) that contained all identified chromosomal ARGs, except for *aadA2b*. This region also included genes encoding IS elements, other functional proteins, proteins of unknown function, and the toxin–antitoxin system (Figures 2A,B). These clustered *dif* sites formed nine *dif* modules, and these modules in a row built an island designated as the “*dif* module island” (Figures 2A,B).

### Plasmid-borne p*dif* sites

We found 19 p*dif* sites in the pM10 of strain M10. Like the configuration of the *dif* module island in the chromosome, 15 p*dif* sites clustered in the region with a size of 25,749 bp (43,812–69,561 bp) that contained all plasmid-borne ARGs and various functional genes (Figures 2A,B). This region was divided into 14 p*dif* modules. Here, we called this region a “p*dif* module island” (Figures 2A,B).

### Xer-ARG modules

**Figure 3:**
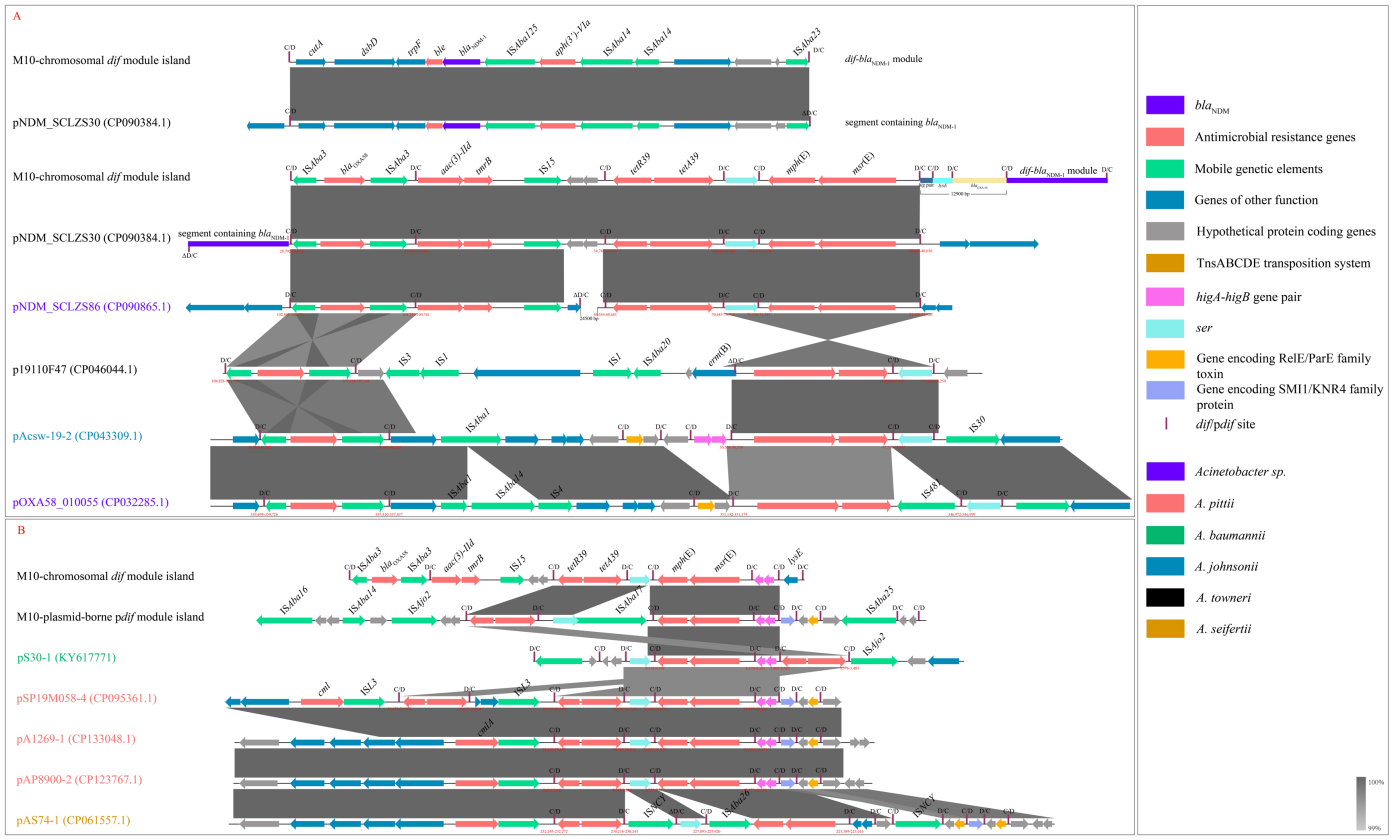
Linear comparisons of the Xer-ARG modules. **(A)** Linear comparisons were built based on the *dif*-*bla*NDM-1, *dif*-*bla*OXA-58, and *dif*-*aac(3)*-*IId* modules. **(B)** Linear comparisons were built based on the Xer-*tet*(39) and Xer-msr(E)-*mph*(E) modules. The C/D and D/C sites of *dif*-ARG and p*dif*-ARG modules are shown in Table 3. The color-marked genome names indicate the species under *Acinetobacter* spp. Vertical bars indicate *dif* or p*dif* (C/D and D/C) sites; the direction of each site is shown above.

In addition to *aadA2b,* the remaining ARGs in the chromosome and plasmid of strain M10 were located within seven Xer modules, including five *dif*-ARG modules (*dif-bla*_OXA-58_, *dif*-*aac(3)-IId, dif-tet*(39), *dif*-*msr*(E)-*mph*(E), and *dif*-*bla*_NDM-1_) in the *dif* module island (Figure 2C) and two p*dif*-ARG modules (p*dif-tet*(39) and p*dif*-*msr*(E)-*mph*(E)) in the p*dif* module island (Figure 2D). The linear alignment in Figure 3A was built based on genome analyses of the *dif*-*bla*_NDM-1_, *dif-bla*_OXA-58_, and *dif*-*aac(3)-IId* modules. The linear alignment in Figure 3B was constructed based on genome analyses of the Xer*-tet*(39) and Xer-*msr*(E)-*mph*(E) modules.

### Genome analyses

#### *Dif*-*bla*_NDM-1_ module

The *Dif*-*bla*_NDM-1_ module, carrying multiple resistance elements (*bla*_NDM-1_, *aph(3′)-*Via, and *ble*_MBL_) and insertion sequences (IS*Aba23*, IS*Aba14,* and IS*Aba125*), was located within the right-hand region of the *dif* module island (Figure 2B). This module, spanning 11,245 bp in size (Figure 2C), was considerably longer than the previous reported (Poirel and Nordmann, 2006; Shao et al., 2023; Merino et al., 2010; Blackwell and Hall, 2017) p*dif*-*bla*_OXA-72_, p*dif*-*bla*_OXA-24_, p*dif*-*bla*_OXA-58_, p*dif*-*sul2*, p*dif-tet*(39) and p*dif*-*msr*(E)-*mph*(E) modules. Notably, the *dif*-*bla*_NDM-1_ module carried the *bla*_NDM-1_ genetic context, IS*Aba125*-*bla*_NDM-1_-*ble*_MBL_-*trpF*-*dsbD*. BLASTn analysis revealed that the *dif*-*bla*_NDM-1_ module exhibited >99% identity and coverage to a segment containing *bla*_NDM-1_ in the pNDM_SCLZS30 (Figure 3A). However, this segment could not be identified as a p*dif* module due to the absence of the right and center regions in the D/C site (Table 3A). Furthermore, the region IS*Aba125*-*bla*_NDM-1_-*ble*_MBL_-*trpF*-*dsbD* in Tn*125* (DANMEL) is highly similar (99.95% identity and 100% coverage) to that in the *dif*-*bla*_NDM-1_ module, but the remaining region of Tn*125*, *cutA*-*groES*-*groEL*-*insE*-IS*Aba125*, is missing in the *dif*-*bla*_NDM-1_ module. No other IS*Aba125* could be identified in the downstream region of the *bla*_NDM-1_ genetic context.

**Table 3:** The C/D and D/C sites of *dif*-ARG and p*dif*-ARG modules in Figure 3.

**(A)** The C/D and D/C sites in Figure 3A							
Xer *bla*_NDM-1_ module	Xer module	C/D			D/C		
		**Left**	**Center**	**Right**	**Left**	**Center**	**Right**
M10_chromosome	*dif* module	GTTTCGTATAA	CAGCCA	TTATGTTAAAT	AATTCACATAA	CATCTC	TTATAAGAATT
pNDM_SCLZS30	p*dif* module	GTTTCGTATAA	CAGCCA	TTATGTTAAAT	TAGTTACATAA	******	***********

Xer *bla*_OXA-58_ module	Xer module	C/D			D/C		
		**Left**	**Center**	**Right**	**Left**	**Center**	**Right**
M10_chromosome	*dif* module	ATTTAACATAA	AGTCTC	TTATACGAAAT	ATTTTGTATAA	GGTGTA	TTATGTTAATT
pNDM_SCLZS30	p*dif* module	ATTTAACATAA	TGGCTG	TTATACGAAAC	ATTTTGTATAA	GGTGTA	TTATGTTAATT
pNDM_SCLZS86		ATTTAACATAA	TGGCTG	TTATACGAAAC	ATTTTGTATAA	GGTGTA	TTATGTTAATT
p19110F47–2		ATTTAACATAA	TGGCTG	TTATACGAAAC	ATTTTGTATAA	GGTGTA	TTATGTTAATT
pAcsw-19-2		ATTTAACATAA	TGGCTG	TTATACGAAAC	ATTTTGTATAA	GGTGTA	TTATGTTAATT
pOXA58_010055		ATTTAACATAA	TGGCTG	TTATACGAAAC	ATTTTGTATAA	GGTGTA	TTATGTTAATT

Xer aac(3)-IId module	Xer module	C/D			D/C		
		**Left**	**Center**	**Right**	**Left**	**Center**	**Right**
M10_chromosome	*dif* module	ATTTTGTATAA	GGTGTA	TTATGTTAATT	ATTGCGTATAA	CCACCA	TTATGTTAAAT
pNDM_SCLZS30	p*dif* module	ATTTTGTATAA	GGTGTA	TTATGTTAATT	ATTGCGTGTAA	CCACCA	TTATGTTAAAT
pNDM_SCLZS86		ATTTTGTATAA	GGTGTA	TTATGTTAATT	***********	******	***********

Xer *tet*(39) module	Xer module	C/D			D/C		
		**Left**	**Center**	**Right**	**Left**	**Center**	**Right**
M10_chromosome	*dif* module	ATTGCGTATAA	CCACCA	TTATGTTAAAT	AATTAACATAA	TACACG	TTATACGAATT
pNDM_SCLZS30	pdif module	ATTGCGTGTAA	CCACCA	TTATGTTAAAT	AATTAACATAA	TACACG	TTATACGAATT
pNDM_SCLZS86		ATTGCGTATAA	CCACCA	TTATGTTAAAT	AATTAACATAA	TACACG	TTATACGAATT

Xer *mph*(E)*-msr*(E) module	Xer module	C/D			D/C		
		**Left**	**Center**	**Right**	**Left**	**Center**	**Right**
M10_chromosome	*dif* module	ACTTCACATAA	GAAATT	TTATGTTAAAT	AATTAACATAA	TACACC	TTATACGAATA
pNDM_SCLZS30	p*dif* module	ACTTCACATAA	GAAATT	TTATGTTAAAT	AATTAACATAA	TACACG	TTATACGAAAT
pNDM_SCLZS86		ACTTCACATAA	GAAATT	TTATGTTAAAT	AATTAACATAA	TACACC	TTATACGAAATA
p19110F47		ACTTCACATAA	GAAATT	TTATGTTAAAT	***********	******	***********
pAcsw-19-2		ACTTCACATAA	GAAATT	TTATGTTAAAT	AATTAACATAA	TACACC	TTATACGAAATA
pOXA58_010055		ACTTCACATAA	GAAATT	TTATGTTAAAT	AATTAACATAA	TACACC	TTATACGAAAT

**(B)** The C/D and D/C sites in Figure 3B							
Xer *tet*(39) module	Xer module	C/D			D/C		
		**Left**	**Center**	**Right**	**Left**	**Center**	**Right**
M10_plasmid	p*dif* module	ATTGCGTATAA	CCACTA	TTATGTTAAAT	AATTAACATAA	TACACG	TTATACGAATT
pS30-1		ATTTCGTATAA	GGTGTA	TTATGTTAATT	ATTTAACATAA	TGGCTG	TTATGCGAAAC
pSP19M058-4-module1		ATTGCGTATAA	CCACTA	TTATGTTAAAT	AATTAACATAA	TACACG	TTATACGAATT
pSP19M058-4-module2		ATTGCGTATAA	CCACTA	TTATGTTAAAT	AATTAACATAA	TACACG	TTATACGAATT
pA1269-1		ATTGCGTATAA	CCACTA	TTATGTTAAAT	AATTAACATAA	TACACG	TTATACGAATT
pAP8900-2		ATTGCGTATAA	CCACTA	TTATGTTAAAT	AATTAACATAA	TACACG	TTATACGAATT
pAS74-1		ATTGCGTATAA	CCACTA	TTATGTTAAAT	AATTAACATAA	TACACG	TTATACGAATT

Xer *mph*(E)*-msr*(E) module	Xer module	C/D			D/C		
		**Left**	**Center**	**Right**	**Left**	**Center**	**Right**
M10_chromosome	*dif* module	ACTTCACATAA	GAAATT	TTATGTTAAAT	AATTAACATAA	TACACC	TTATACGAATA
M10_plasmid	p*dif* module	ACTTCACATAA	GAAATT	TTATGTTAAAT	AATTAACATAA	TACACC	TTATACGAAAT
pS30-1		ACTTCACATAA	GAAATT	TTATGTTAAAT	ATTTAACATAA	TACACC	TTATACGAAAT
pSP19M058–4		ACTTCACATAA	GAAATT	TTATGTTAAAT	AATTAACATAA	TACACC	TTATACGAAAT
pA1269-1		ACTTCACATAA	GAAATT	TTATGTTAAAT	AATTAACATAA	TACACC	TTATACGAAAT
pAP8900-2		ACTTCACATAA	GAAATT	TTATGTTAAAT	AATTAACATAA	TACACC	TTATACGAAAT
pAS74-1		ACTTCACATAA	GAAATT	TTATGTTAAAT	AATTAACATAA	TACACC	TTATACGAAAT

The highlighted bases in red are different from the chromosomal *dif* sites of strain M10. The highlighted bases in green indicate the insertion of a base. The highlighted * in red is the absence of bases. The C/D and D/C are a 28 bp site consisting of two inverted repeat 11 bp XerC and XerD binding motifs (the left and the right regions) separated by a six bp interval (central region).

#### *Dif-bla*_OXA-58_ module

Two inverted *bla*_OXA-58_ genes were identified within the different modules in the *dif* module island (Figure 2B). These *bla*_OXA-58_ genes were flanked by two inverted IS*Aba3* elements, with sizes 427 bp and 774 bp, respectively. These IS elements were shorter than the full-length IS*Aba3* element (794 bp, ISFinder). The right-hand *dif*-*bla*_OXA-58_ module (10,747 bp) shared the D/C and C/D sites of the *dif*-*lysE* and *dif*-*bla*_NDM-1_ modules. The analysis in GenBank databases found that no segment matches this module. The left-hand *dif*-*bla*_OXA-58_ module (2,318 bp) shared the D/C site with the *dif*-*aac(3)-IId* module. BLASTn analysis revealed multiple segments of *Acinetobacter* plasmids exhibiting >99% identity and coverage with this module. Thus, this study focuses on the left-hand *dif*-*bla*_OXA-58_ module. All segments containing *bla*_OXA-58_ in Figure 3A could be identified as p*dif*-*bla*_OXA-58_ modules. These modules were highly conserved (>99% identity and coverage) with the chromosomal *dif*-*bla*_OXA-58_ module. Four variant bases emerged in the center and right regions of the C/D site within p*dif* sites compared to *dif* sites (Table 3A).

#### *dif*-*aac(3)-IId* module

The *dif*-*aac(3)-IId* module of 3,736 bp carrying *aac(3)-IId*, *tmrB*, and IS*15* (Figure 2C) was positioned between the *dif-bla*_OXA-58_ and *dif-tet*(39) modules, sharing their C/D and D/C sites (Figure 2B). BLASTn analysis revealed two plasmid-borne fragments (pNDM_SCLZS30 and pNDM_SCLZS86) exhibiting >99% identity and coverage with the *dif*-*aac(3)-IId* module (Figure 3A). In pNDM_SCLZS86, the downstream region of the segment containing *aac(3)-IId* was truncated by a 24,500-bp region, leading to the absence of D/C site (Table 3A). P*dif*-*aac(3)-IId* module in pNDM_SCLZS30 could be identified and was highly conserved (>99% identity and coverage) with the chromosomal *dif*-*aac(3)-IId* module. Only one variant base emerged in the left region of the D/C site within p*dif* sites compared to *dif* sites (Table 3A).

#### Xer*-tet*(39) modules

Xer-*tet*(39) modules were identified in the *dif* and p*dif* module islands, respectively (Figures 2B–D). These modules exhibited >99.5% identity and coverage (2056/2057 bp) (Figure 3B). BLASTn analysis revealed they showed high similarity (>99% identity and coverage) to numerous segments of the chromosomes and plasmids of *Acinetobacter* in the public database. Eight p*dif*-*tet*(39) modules could be identified from public plasmid genomes (Figure 3), two of which were identified in pSP19M058–4. Notably, the p*dif*-*tet*(39) module in pS30-1 has been previously reported (Blackwell and Hall, 2017). It differed from the chromosomal *dif-tet*(39) module in the center regions of the C/D and D/C sites (Table 3B). The remaining modules were highly conserved (>99% identity and coverage) to chromosomal *dif-tet*(39) module (Figure 3). Only one variant base emerged in the center region of C/D site within p*dif* sites compared to *dif* sites (Tables 3A,B).

#### Xer*-msr*(E)-*mph*(E) modules

Xer-*msr*(E)-*mph*(E) modules were located in the *dif* and p*dif* module islands, respectively (Figures 2B–D). These modules exhibited >99.5% identity and coverage (3,004/3006 bp). Like the *dif*-*tet*(39) module, they were also highly similar (>99% identity and coverage) to numerous segments of the chromosomes and plasmids of *Acinetobacter* in the public database. In addition to the segment in p19110F47, nine p*dif*-*msr*(E)-*mph*(E) modules could be identified from public plasmid genomes (Figure 3). Interestingly, the p*dif*-*msr*(E)-*mph*(E) modules in pOXA58_010055 and pAS74-1 additionally carried IS*481* and IS*Aba26*, respectively. Apart from these modules, the remaining modules were highly similar (>99% identity and coverage) to the chromosomal *dif*-*msr*(E)-*mph*(E) module (Figure 3). Compared to *dif* sites, two variant bases or one inserted base emerged in the right region of the D/C site within p*dif* sites (Tables 3A,B).

#### Chromosomal *dif* modules containing other genes

In addition to ARGs, three *dif* modules containing other genes have been identified in the *dif* module island. The *dif*-*ser* module (2,537,075-2,537,939 bp) was located between the *dif*-*tet*(39) and *dif*-*msr*(E)-*mph*(E) modules, sharing their D/C and C/D sites (Figure 2B). This gene encoded a helix-turn-helix domain-containing protein (UniProt, 203 aa), which is a small serine recombinase and belongs to the site-specific recombinase resolvase family. The copy of this gene could be identified in the plasmid-carried p*dif*-*ser-*IS*Aba17* module in pM10. Interestingly, the Xer-*ser* module could be frequently identified in public plasmid genomes and was typically located in the downstream region of *mph*(E) (Figure 3). The *dif*-*higA*-*higB* module (2,540,890-2,541,651 bp) was located between *dif*-*msr*(E)-*mph*(E) and *dif*-*lysE* modules and shared their D/C and C/D sites (Figure 2B). The *higB* encoded a stable toxin protein (106 aa) and the *higA* encoded its labile antitoxin (90 aa). These genes belong to the type II toxin–antitoxin system (a critical genetic regulatory module in bacterial plasmids), which maintains plasmid stability by inhibiting growth when the plasmid is lost, and is widely distributed across bacterial plasmids and chromosomes. This module was highly similar (>99% identity and coverage) to the p*dif*-*higA*-*higB* module (58,052–58,809 bp) identified in the p*dif* module island (Figure 2B) and public plasmid genomes (Figure 3). Furthermore, the *dif* module (2,541,624–2,543,218 bp, Figure 2B), harboring a similar gene (99.5% identity) to *lysE* family translocators (201 aa, bacterial transmembrane transporters specialized in amino acid efflux to maintain intracellular homeostasis), was located between *dif*-*higA*-*higB* and *dif*-*bla*_OXA-58_ modules and shared their C/D and D/C sites (Figure 2B). This module was undetectable in pM10 and public plasmid genomes (Figure 3).

#### Plasmid-borne p*dif* modules containing other genes

In addition to ARGs, multiple p*dif* modules carrying other genes were detected in the p*dif* module island. A similar gene (99.5% identity) to STAS domain-containing proteins (485 aa, a conserved structural domain ubiquitously present in prokaryotic and eukaryotic organisms) was detected in two *dif* modules (43,812–45,969 and 50,114–52,850 bp), respectively (Figure 2B). The STAS domain is functionally implicated in diverse cellular processes, particularly mediating ion transport and signal transduction. The p*dif* module (56,834–57,585 bp) contains a functionally uncharacterized gene and a gene encoding RelE/ParE family toxin (100 aa) of the Type II toxin–antitoxin system (Figure 2B). Adjacent to this module, a p*dif* module (57,558–58,080) carried a gene encoding a SMI1/KNR4 family protein (139 aa), a functionally unknown protein in bacterial systems (Figure 2B). Notably, the above two modules were commonly present across public plasmid genomes (Figure 3B). In addition, three p*dif* modules, including 48,072–49,400 bp, 49,373–50,141 bp, and 52,823–53,701 bp, were identified in the p*dif* module island and carried multiple uncharacterized genes (Figure 2B).

#### p*dif*-IS*Ajo2* module

In the p*dif* module island, two highly similar IS elements were identified in the p*dif* modules of 45,942–48,099 bp and 67,207–69,561 bp and were 1,482 bp (left-hand module) and 1,475 bp (right-hand module) (Figure 2B). They showed 97% identity to the IS*Ajo2* reference sequence, 96% identity to IS*Aso2*, and 90% identity to IS*Api2* (ISFinder). Given its highest similarity to IS*Ajo2* (an IS*Aba32* group member of the IS*1202* family), this element is designated as IS*Ajo2* (ISfinder). Notably, the IS*Ajo2* in the left-hand module was five bp from the C/D site. This characteristic was similarly observed in previous reports (Blackwell and Hall, 2017; Harmer et al., 2023). However, two additional genes, *relB* (81 aa) *and relE* (83 aa), were identified downstream of IS*Ajo2* in this module (Figure 2B). These genes encode functional components of the family addiction module antitoxins and constitute essential elements within bacterial toxin–antitoxin systems. The IS*Ajo2* in the right-hand module was 27 bp from the C/D site. Two genes encoding a BrnT-family toxin (103 aa) and a cytoplasmic protein (104 aa) were identified downstream of the IS*Ajo2* (Figure 2B). However, the characteristics of this module were different from the previous reports (Blackwell and Hall, 2017; Harmer et al., 2023).

#### p*dif*-IS*Aba25* module

A previously unreported IS in the Xer module was identified in the p*dif* module (53,674–56,861 bp, Figure 2B) of the p*dif* module island. Sequence analysis found that this element exhibited 95% identity to the IS*Aba25* reference sequence and 93% identity to IS*Alw34*. Based on its best match, it was designated as IS*Aba25* (ISfinder), a member of the IS*66* family. Furthermore, a gene encoding an uncharacterized protein (170 aa) was detected downstream of this IS.

#### p*dif*-IS*Aba17* module

Another previously unreported IS in the Xer module was identified in the p*dif* module (61,760–65,205 bp, Figure 2B) of the p*dif* module island. This element exhibited 95% identity to the reference sequence IS*Aba17* (ISfinder), a member of the IS*66* family. A gene that was similar to the *ser* gene in the *dif* module (2,537,075–2,537,939 bp) was detected upstream of this IS. However, its 102 bases at the 3′ end were replaced by IS*Aba17* (Figure 2B).

#### The configuration of the *dif* and p*dif* module islands

Xer-*msr*(E)-*mph*(E) module was closely adjacent to Xer-*tet*(39) module in the *dif* and p*dif* module islands (Figure 2B). This characteristic was also frequently observed in public plasmid genomes (Figure 3). Similarly, *dif*-*bla*_OXA-58_ module was closely adjacent to *dif*-*aac(3)-IId* module, and this characteristic also appeared in some public plasmid genomes (Figure 3A). The *dif* and p*dif* module islands were formed by multiple *dif*/p*dif* modules arranged in a row. This configuration inevitably leads to sharing the internal C/D or D/C sites to form two types of *dif* modules: one flanked by a C/D and a D/C site and another flanked by a D/C and a C/D site (Figure 2B). A similar configuration was also frequently observed in public plasmid genomes (Figure 3).

## Discussion

*Acinetobacter*, a heterogeneous bacterial genus, is widely distributed across human and animal communities and exhibits robust environmental adaptability and survival capabilities (De Amorim and Nascimento, 2017). Many of them, primarily *A. baumannii,* but also *A. nosocomialis*, *A. pittii*, *A. lwoffii*, and others, are listed as clinically relevant pathogens (Wang et al., 2025; Touchon et al., 2014; Peleg et al., 2012). The rising antimicrobial resistance, particularly resistance to last-resort antibiotics, among this genus has become an urgent medical challenge, with numerous mobile genetic elements contributing to its dissemination. A presumptive p*dif*-*bla*_OXA-24_ module was initially identified in the *A. baumannii* plasmid pABVA01a (D’Andrea et al., 2009). This module was also found in different contexts within another *Acinetobacter* plasmid, suggesting its transfer (D’Andrea et al., 2009). Subsequent studies revealed that *Acinetobacter* plasmids carry p*dif* modules with a wide variety of resistance genes, metal resistance genes, IS elements, and other genes (Wang et al., 2025; Grosso et al., 2012; Midonet and Barre, 2014; Shao et al., 2023; Cameranesi et al., 2018; Merino et al., 2010; Blackwell and Hall, 2017; Tran et al., 2012; Harmer et al., 2023; Mindlin et al., 2019). Thus, these modules were previously considered to be associated with *Acinetobacter* plasmids. Although additional *dif*-like sites have been identified on the chromosome of *Acinetobacter* beyond the main locus (Mindlin et al., 2019), it remains to be determined whether these sites are associated with the *dif* module. This study revealed that multiple Xer sites are present in the chromosome and plasmid of *Acinetobacter* sp. M10, mediating the formation of simple Xer modules carrying one to several genes or complex Xer modules containing more than ten genes. Genome analysis confirmed that the *dif-tet*(39) and *dif*-*msr*(E)-*mph*(E) modules in the chromosome were highly similar (sequence identity >99%) to those in the plasmid of this strain. Multiple chromosomal *dif* modules in this strain showed high sequence identity (>99%) to plasmid-carried p*dif* modules in public databases. Genome analysis and antimicrobial susceptibility testing confirm the association between these modules and the resistant phenotype. These suggest that *Acinetobacter* chromosomes also function as a vehicle for *dif* modules, and various genes (such as genes encoding antibiotic resistance, IS elements, toxin–antitoxin systems, and other functional proteins) flow via Xer modules between the chromosomes and plasmids of *Acinetobacter*. These modules, arranged one after another in the chromosome and plasmid, form special configurations: a chromosomal *dif* module island and a plasmid-borne p*dif* module island, suggesting their presence either as single modules or linear multi-module arrays. Similar configurations were frequently observed in public *Acinetobacter* plasmid genomes. The exploration of the chromosomal *dif* modules and the plasmid-carried p*dif* modules provides a novel perspective for understanding the dissemination of antibiotic-resistant *Acinetobacter* worldwide.

The recurrent appearance of Xer modules on the chromosomes and plasmids of *Acinetobacter* demonstrates their ability to spread. Like other MGEs, Xer modules mediate the horizontal transfer of genes, accelerating bacterial genome evolution. The Xer module itself functions as a “gene cassette” that can be integrated into larger genetic contexts, such as plasmids and chromosomes (Shao et al., 2023). When such a plasmid transfers among bacterial populations via conjugation, the Xer module “hitchhikes” along, reflecting the synergistic mechanism among MGEs. The transfer mechanism of Xer modules shares common characteristics with other MGEs while exhibiting specific characteristics. Like the transfer of other canonical MGEs (such as plasmids, ICEs, and transposons) (Wang et al., 2025; Correa et al., 2024; Bi et al., 2012; Brovedan et al., 2020), which depend on various elements (such as *oriT*, relaxase, Rep, and transposases), the Xer module relies on the XerCD site-specific recombination system. However, unlike other MGEs, the Xer module encodes only Xer sites and does not encode other self-transfer elements (Shao et al., 2023; Blackwell and Hall, 2017). Its mobilization usually depends on the mechanism provided by the host cell. These features give the Xer module a more compact structure and potentially reduce the burden on the host bacteria. As a novel gene dissemination system, Xer modules facilitate the spread of antibiotic resistance within the *Acinetobacter* genus, together with other MGEs, forming a complex and dynamic network of gene flow.

Previous studies (Mindlin et al., 2019; Blakely et al., 1993; Blakely et al., 1991; Kuempel et al., 1991) found that a single main *dif* site in the terminus region of the chromosome is required for the resolution of chromosome dimers by the XerC/XerD system. If it is transferred, the dimer resolution process is impaired (Balalovski and Grainge, 2020; Kuempel et al., 1991). Numerous *dif* sites identified in the chromosome of *Acinetobacter* sp. M10 suggest that additional *dif*-like sites are present in the chromosome of *Acinetobacter* beyond the main locus and unlikely to play the function of dimer resolution. Genome analysis revealed that *dif* sites and associated *dif* modules were highly similar (sequence identity >99%) to p*dif* sites and associated p*dif* modules, suggesting that additional chromosomal *dif*-like sites facilitate *dif* module formation. The results of this study and previous studies (Blackwell and Hall, 2017; Mindlin et al., 2019; D’Andrea et al., 2009; Feng et al., 2016) collectively highlight the common presence of the Xer site and associated Xer module across plasmids and chromosomes within the *Acinetobacter* genus. The XerCD site-specific recombination system, initially functioning to stabilize chromosomes and plasmids (Balalovski and Grainge, 2020; Carnoy and Roten, 2009; Castillo et al., 2017), has subsequently played an additional role in driving horizontal gene transfer, underscoring that bacterial evolution never ceases. This study identified a chromosomal *dif* module island and a plasmid-borne p*dif* module island. These islands consist of multiple *dif*/p*dif* modules arranged one after another, leading to modules flanked by a C/D and a D/C sites or by a D/C and a C/D sites. This characteristic raises the question of whether the horizontal mobilization of the *dif/pdif* modules occurs as an individual module or as a linearly contiguous multi-module array.

The *bla*_NDM_ gene encodes New Delhi Metallo-*β*-lactamase (NDM), which hydrolyzes nearly all β-lactam antibiotics, including carbapenems, the last-resort therapeutic agents for multidrug-resistant bacterial infections (Kikuchi et al., 2022; Wang et al., 2017; Acman et al., 2022; Chatterjee et al., 2016; Partridge and Iredell, 2012). The *bla*_NDM_-positive strains were first retrospectively identified in 2005 from *A. baumannii* in an Indian hospital (Kikuchi et al., 2022; Acman et al., 2022; Partridge and Iredell, 2012). In early isolates, *bla*_NDM_ is located within the intact Tn*125* transposon, leading to the hypothesis that Tn*125* is the ancestral transposon of *bla*_NDM_ (Acman et al., 2022). The upstream region of Tn*125* carries ISs from families such as IS*5*/IS*30*, and frequent recombination among these IS elements has generated diverse genetic backgrounds (Kikuchi et al., 2022; Acman et al., 2022). IS*Aba125*, a member of the IS*30* family, is typically located upstream of *bla*_NDM_ and forms the structure IS*Aba125*-*bla*_NDM_-*ble*_MBL_-*trpF*-*dsbD* (Acman et al., 2022). This structure was frequently detected in *Acinetobacter*, *Klebsiella*, and *Escherichia* (Acman et al., 2022). It has been widely accepted that *bla*_NDM-1_ is regulated by a hybrid promoter located between *bla*_NDM-1_ and IS*Aba125*, and IS*Aba125* is commonly present in some form within *bla*_NDM_-positive isolates (Kikuchi et al., 2022). This study identified an intact *bla*_NDM-1_ genetic backbone carried by the chromosomal *dif* module, suggesting that, for effective expression of carbapenem resistance, *bla*_NDM-1_, closely associated elements, and the hybrid promoter are integrally preserved in *the dif* module.

This study identified multiple Xer modules carrying genes encoding toxin–antitoxin systems, IS elements, helix-turn-helix domain-containing proteins, and proteins of uncharacterized function. Genome analyses confirmed that copies of these modules were widely distributed in *Acinetobacter* genomes. Given that genes in Xer modules are frequently associated with toxin–antitoxin systems, antibiotic resistance, and metal resistance, the Xer modules may contribute to the persistence of their resident vehicles, such as plasmids or chromosomes, by enhancing adaptability and tolerance. Various Xer modules were detected in the chromosome and plasmid of *Acinetobacter* sp. M10, which may suggest the presence of numerous undiscovered Xer modules within the *Acinetobacter* population. Furthermore, numerous *dif* and p*dif* modules detected in a novel species of the *Acinetobacter* genus underscore the potential for widespread dissemination of ARGs and mobile elements in this genus.

## Conclusion

Additional chromosomal *dif*-like sites are functionally similar to plasmid-carried p*dif* sites. Modules flanked by Xer sites, as a novel mobile genetic element, form a close interaction network with their vehicles, such as plasmids and chromosomes, and play a vital role in disseminating resistance genes and other functional elements.

## Materials and methods

### Sampling and microbial identification

Strain M10 was collected from the feces of a farmer at a commercial bovine farm in Guangxi, China. Human-derived specimens were obtained following written informed consent procedures. This strain was isolated from MacConkey (Huan Kai Microbial, China) agar plates supplemented with 0.5 mg/mL meropenem and cultured at 37 °C for 16 h. As described previously (Wang et al., 2017), PCR amplification and Sanger sequencing were used to detect the *bla*_NDM_ gene.

### Whole-genome sequencing

Briefly, the genome was sequenced using the long reads on the Oxford Nanopore platform (Beijing Genomics Institution, China) and the short reads on the DNBSEQ platform (Beijing Genomics Institution, China). The corrected reads were obtained by hybrid assembly of DNBSEQ and Oxford Nanopore. The assembled genome was checked for completeness and contamination using CheckM (v1.2.3) of the NCBI annotation service.

### Bacterial-species identification

Bacterial genus identification was performed using matrix-assisted laser desorption/ionization time-of-flight mass spectrometry (Singhal et al., 2015) (MALDI-TOF MS; Bruker Daltonik, Germany) and 16 s rDNA (Kim et al., 2010). Bacterial species identification was carried out on the NCBI database using the ANI match of the NCBI annotation service, on the JSpeciesWS platform (JSpeciesWS – Taxonomic Thresholds) using TCS, and on the DSMZ platform (Type Strain Genome Server) using TYGS. The dDDH value was calculated using GGDC on the DSMZ platform (Leibniz Institute DSMZ: Welcome to the Leibniz Institute DSMZ).

### Phylogenetic construction

Core genomes were extracted using Roary (Roary). Recombination was filtered using Gubbins (Gubbins). Filtered polymorphic sites were employed to build a tree on the PhyML platform (PhyML). The iTOL (Interactive Tree Of Life) was used to visualize the tree based on each genome’s features.

### Antimicrobial susceptibility testing

Minimum inhibitory concentrations (MICs) were determined for 18 antibiotics, listed in Table 1, using the broth microdilution method in Mueller–Hinton medium (HuanKai Microbial, China). Interpretive criteria of Clinical and Laboratory Standards Institute (CLSI) guidelines (M100-S34, 2024) were applied. The CLSI breakpoints for *Enterobacteriaceae* were used for aztreonam, chloramphenicol, kanamycin, fosfomycin, azithromycin, and ertapenem, as criteria for *Acinetobacter* were unavailable. The resistance breakpoints for *Enterobacterales* set by the U. S. Food and Drug Administration (FDA) were used to interpret tigecycline (R ≥ 8 mg L^−1^) as the CLSI criteria for this antimicrobial, and the FDA criteria for *Acinetobacter* were absent.

### Bioinformatics analysis

The MOB-typer on the Galaxy platform (Galaxy | China) was used to analyze plasmid characteristics. The VFanalyzer on the VFDB platform (VFDB: Virulence Factors of Bacterial Pathogens) was used to search for genes encoding VFs. Genome annotation was performed using the RAST genome annotation service (RAST Server – RAST Annotation Server), and further manual correction was performed using ORFfinder (ORFfinder Home-NCBI), UniProt (UniProt), and ISFinder (ISfinder). The linear alignment was created using Easyfig (v2.2.5).

### The identification of *dif* and p*dif* sites

Identification of *dif* and p*dif* sites was performed using BLAST tools. p*dif* sites were identified according to the p*dif* sites of *A. baumannii* and *A. lwoffii* in the previous report (Mindlin et al., 2019). *dif* sites were identified based on the chromosomal additional *dif*-like sites of *Acinetobacter* in the previous report (Mindlin et al., 2019). Only sites that were at least 85% identical to the reference sequences were taken into account.

## Data Availability

The assembled genome of strain M10 has been deposited in the NCBI database under accession no. GCA_048572845.1.
